# Asexual Reproduction Does Not Apparently Increase the Rate of Chromosomal Evolution: Karyotype Stability in Diploid and Triploid Clonal Hybrid Fish (Cobitis, Cypriniformes, Teleostei)

**DOI:** 10.1371/journal.pone.0146872

**Published:** 2016-01-25

**Authors:** Zuzana Majtánová, Lukáš Choleva, Radka Symonová, Petr Ráb, Jan Kotusz, Ladislav Pekárik, Karel Janko

**Affiliations:** 1 Laboratory of Fish Genetics, Institute of Animal Physiology and Genetics, CAS, v.v.i, Liběchov, Czech Republic; 2 Department of Zoology, Faculty of Science, Charles University in Prague, Prague, Czech Republic; 3 Department of Biology and Ecology, Faculty of Science, University of Ostrava, Ostrava, Czech Republic; 4 Research Institute for Limnology, University of Innsbruck, Mondsee, Austria; 5 Museum of Natural History, University of Wrocław, Wrocław, Poland; 6 Institute of Botany, SAS, Bratislava, Slovakia; 7 Department of Biology, Faculty of Education, Trnava University, Trnava, Slovakia; Ecole normale superieure de Lyon, FRANCE

## Abstract

Interspecific hybridization, polyploidization and transitions from sexuality to asexuality considerably affect organismal genomes. Especially the last mentioned process has been assumed to play a significant role in the initiation of chromosomal rearrangements, causing increased rates of karyotype evolution. We used cytogenetic analysis and molecular dating of cladogenetic events to compare the rate of changes of chromosome morphology and karyotype in asexually and sexually reproducing counterparts in European spined loach fish (*Cobitis*). We studied metaphases of three sexually reproducing species and their diploid and polyploid hybrid clones of different age of origin. The material includes artificial F1 hybrid strains, representatives of lineage originated in Holocene epoch, and also individuals of an oldest known age to date (roughly 0.37 MYA). Thereafter we applied GISH technique as a marker to differentiate parental chromosomal sets in hybrids. Although the sexual species accumulated remarkable chromosomal rearrangements after their speciation, we observed no differences in chromosome numbers and/or morphology among karyotypes of asexual hybrids. These hybrids possess chromosome sets originating from respective parental species with no cytogenetically detectable recombinations, suggesting their integrity even in a long term. The switch to asexual reproduction thus did not provoke any significant acceleration of the rate of chromosomal evolution in *Cobitis*. Asexual animals described in other case studies reproduce ameiotically, while *Cobitis* hybrids described here produce eggs likely through modified meiosis. Therefore, our findings indicate that the effect of asexuality on the rate of chromosomal change may be context-dependent rather than universal and related to particular type of asexual reproduction.

## Introduction

Most species differ by their karyotype, which are routinely defined by the variation in number, size and morphology of chromosomes. Differences originating during karyotype evolution play an important role in speciation [1]. Simultaneously, the rate at which chromosome rearrangements accumulate varies greatly from one species to another. For example, changes in chromosome number have occurred roughly 20 times faster in mammals than in frogs [2] (although even there one can find lineages with exceptionally fast rate of chromosomal evolution, e.g. [3]). With more than 27.000 taxonomically known species [4], teleost fishes show rather conserved karyotypes with a uniform haploid chromosome number n = 24–25 or close to it [5,6], suggesting a slow rate of karyotype change, mostly by intrachromosomal rearrangements [7]. However, a number of exceptions exist, especially in lineages of polyploid origin (see [8]).

The reasons why karyotypes remain conservative in some groups while they readily mutate and rearrange in others continue to be the focus of ongoing and lively debate (e.g. [9]). At least some factors have been proposed to modify rates of karyotype evolution. One of them, polyploidization, has been shown to instantaneously increase the chromosome numbers [10] but also to directly increase rates of chromosome breakages and aneuploidy incidence [11]. Asexuality (defined in a broader sense by the lack of efficient recombination and segregation; e.g. [12]) is another factor that is supposed to significantly influence the rate of chromosomal changes [13]. Relaxed constraints on the pairing of homologous chromosomes during gamete formation in clones may allow faster accumulation of chromosomal mutations than in sexual species (e.g. [14]). An absence of effective recombination ultimately leads to an accumulation of independent mutations on any locus including whole chromosomes (Meselson effect; [15]). Chromosomal complements of asexual organisms, where most of them are also polyploids, may therefore acquire strongly aneuploid or structurally changed characteristics relatively easily. The hypothesis of asexuality-linked increase in the rate of karyotype evolution is supported by studies of apomictic arthropods [13,14,16–18] and root knot nematodes [19] which suggest that once sex is lost, chromosomal structure can become irregular extremely quickly; e.g. chromosomal fissions in *Sitobion miscanthi* aphids occurred over several dozens of generations during seven years in laboratory culture [13].

However, number of uncertainties remains. Especially, it is unclear whether the aforementioned indications of increased rates of karyotype changes could be generally attributed to asexual reproduction *per se* because other features of those organisms, especially the holokinetic nature of their chromosomes, may also influence resulting patterns [20]. Moreover, since abovementioned studies were focused on apomictic parthenogens (i.e. with eggs formed by mitosis) it is unknown whether other types of asexual reproduction have the same effect on the rate of morphological change of karyotype. Actually, asexuality can be achieved by many cytological mechanisms, which have profoundly different predictions about the heterozygosity of resulting individuals [21,22]. This may affect the rate of chromosomal evolution differently [14]. Certain types of automixis may be exactly equivalent to apomixis [23], e.g. premeiotic endoduplication. In that case, the genetic material is duplicated before entering meiosis while segregation and recombination take place on bivalents that form between sister chromatids, the genetically identical copies derived from endoduplication event. The progeny is therefore genetically identical to its mother [24]. Other types of automixis may lead to intragenomic recombination or exclusions of large genomic parts (e.g. [25]). Therefore, it is important to test the general applicability of the hypothesis of asexuality-driven increase of karyotype mutation rate.

Spined loaches from the *Cobitis taenia* hybrid complex (*sensu* Janko *et al*. [26]) are fish model with included automictic reproduction, allowing us to study karyotype changes in vertebrate automicts as an alternative to published cases from invertebrate apomicts. Primary hybridization between *C*. *elongatoides* and either *C*. *taenia* or *C*. *tanaitica* (two sister species distantly related to *C*. *elongatoides*; [27]) leads to diploid hybrid clonal lineages reproducing via gynogenesis. In this reproductive mode, the sperm-dependent parthenogenetic females produce unreduced eggs that are only activated but not truly fertilized by sperm from males of one of the parental species. Therefore, males serve as hosts for these so called clonal sexual parasites [28–30]. Clonal spined loaches have retained meiosis [31] but unreduced egg production is ensured by premeiotic genome doubling [32], a similar mechanism to the clonal pond loach *Misgurnus anguillicaudatus* [33]. Interestingly, having originated as diploid hybrids, these lineages expanded over large parts of the European continent (for distribution of particular *Cobitis* hybrid population types in Europe see [34,35]). They also largely tend to increase the ploidy level by accidental incorporation of sperm nucleus into unreduced clonal eggs at fertilization via genome addition, resulting in polyploid (mostly 3n) clonal lineages [26,28,29].

Considering that interspecific hybridization with subsequent clonal formation has been proceeding continuously at least since the last interglacial [26], contemporary clonal assemblages represent a mixture of very recent as well as relatively old clonal hybrid lineages. The oldest asexual lineage (referred to as the ‘Hybrid clade I’) apparently predates the last glacial maximum and its age was estimated to be roughly 0.3 MYA [26].

In the present study we compare rates of morphological evolution of karyotypes of three sexual species (*C*. *elongatoides*, *C*. *taenia* and *C*. *tanaitica*) and their wild-caught diploid and triploid hybrid biotypes with asexual reproduction. Hybrid biotypes differ by age represented by three classes: F1 laboratory-produced clonal hybrids; individuals derived from recent (Holocene) hybridizations; individuals derived from ancient (interglacial) hybridizations. Using conventional (Giemsa staining) and molecular (genomic *in situ* hybridization; GISH) cytogenetic methods we test following tasks: i) whether visible changes in chromosome number and/or morphology occurs in karyotypes of hybrid biotypes; ii) whether the original parental chromosomal sets can be unambiguously identified in hybrid karyotypes of clones of different age and iii) whether large-scale recombinations and/or rearrangements can be detected. Karyological observations were put into phylogenetic and time-based context with a newly dated phylogeny of parental species as well as hybrids by using available calibration point. This point was studied both on a relative and absolute time scales by visualizing the changes in karyotypes of parental species onto dated phylogeny. The dating allowed testing of the hypothesis whether asexual reproduction and/or hybridity have notably increased the rate of chromosomal changes in comparison with karyologically diversified sexual lineages. We particularly focused on whether karyotypes of older clonal lineages differ from contemporary parental species more than those recently derived.

## Materials and Methods

### Background knowledge about karyotypes of parental species

Sexual parental species included in this study are *C*. *elongatoides*, *C*. *taenia* and *C*. *tanaitica* and hereafter we use the acronyms E, T and N for their haploid genomes, respectively. These are used to designate species and also hybrids with various genomic combinations (e.g. triploid hybrid biotype containing two haploid genomes of *C*. *elongatoides* and one haploid genome of *C*. *tanaitica* is encoded EEN). To evaluate rates of karyotype evolution in sexual species and hybrid clones, we take an advantage of previous karyological studies of European spined loaches. While no intraspecific karyotype differences has been discovered so far, it has been shown that karyotypes of parental species have diverged from each other by various chromosomal rearrangements involving one probable fusion and numerous pericentric inversions [34,36]. In contrast with the substantial karyotype differentiation among parental species, several studies demonstrated consistency of karyotypes within species across their distribution ranges [34,36–38]. Multilocus phylogenetic study [27] suggested the sister relationship of sexual *C*. *taenia* and *C*. *tanaitica* species with *C*. *elongatoides* being their distant relative. In concordance with this hypothesis, *C*. *elongatoides* (2n = 50) possesses the most differentiated karyotype dominated by meta- and submetacentric chromosomes, while karyotypes of *C*. *tanaitica* and *C*. *taenia* are more similar to each other and dominated by subtelo- and acrocentric chromosomes. They differ, however, in number of chromosomes and their centromere positions with *C*. *taenia* (2n = 48) having more acrocentrics than *C*. *tanaitica* (2n = 50). A detailed karyotype characteristic of sexual species is given in Table 1.

**Table 1:** Karyotype characteristics of *Cobitis* genomes with various ploidy levels and genomic compositions of biotypes involved in this study.

ploidy level	(n)			(2n)					(3n)			
genome composition	E	T	N	EE	TT	NN	ET	EN	EET	ETT	EEN	ENN
metacentric chromosomes	11	5	5	22	10	10	16	16	27	21	27	21
submetacentric chromosomes	13	9	13	26	18	26	22	26	35	31	39	39
subtelocentric chromosomes	1	1	3	2	2	6	2	4	3	3	5	7
acrocentric chromosomes	-	9	4	-	18	8	9	4	9	18	4	8
total number of chromosomes	25	24	25	50	48	50	49	50	74	73	75	75

Abbreviations: Capital letters represent sets of haploid genomes: E, *Cobitis elongatoides*; T, *C*. *taenia*; N, *C*. *tanaitica*.

### Animals used in this study

Altogether 57 *Cobitis* individuals of nine biotypes were used in the study (Table 2). The examined animals included di- and triploid clonal forms of various genomic constitutions, i.e. EN, ET, EEN, ENN, EET and ETT. Since the genomic composition of species and hybrid forms may not be identified by external morphology because of their cryptic nature, the genome of each individual was determined by species-specific allozyme markers (*Aat*, *Mdh*, *Pgm*, *Gpi-A* and *Ldh*) according to Janko *et al*. [28]. Interspecific hybrids mostly came from native populations in Europe from Bulgaria (2 localities, 4 individuals), Germany (3 localities, 4 individuals), Poland (2 localities, 10 individuals), Romania (4 localities, 18 individuals), Slovakia (1 locality, 8 individuals) and Czech Republic (1 locality, 1 individual). In addition, one laboratory-born first-generation (F1) ET and three backcrossed (B1) ETT hybrids were analysed as control specimens to test the karyotype stability and to standardize the GISH approach for correct classification of chromosomes between hybrid and parental complements. The maternal ancestry of a subset of studied hybrid individuals and their divergence from parental species were determined using mitochondrial cytochrome *b* gene (cyt *b*) according to Janko *et al*. [30].

**Table 2:** Individuals used in this study.

Biotype	Country	Locality	Lat	Long	AOC	NOI	NOMKA	NOGE	NOAGM
**EE**	Hungary	Szodrakosz Cr.	47°43'58.8"N	19°07'58.8"E	-	1	5	-	-
	Poland	Budkowiczanka R.	50°50'50.1"N	18°11'07.1"E	-	1*	-	-	-
**TT**	Germany	Haaren Cr.	53°04'58.8"N	7°49'58.8"E	-	2* + 1	4	-	-
**NN**	Romania	Danube R.	44°04'47.9"N	26°43'51.2"E	-	2*	-	-	-
	Romania	Sinoe at Histria	44°37'58.8"N	28°52'58.8"E	-	1	4	-	-
**ET**	Czech Rep.	Laboratory F1	50°24'37.6"N	14°27'16.9"E	F1 generation	1	9	1	4
	Poland	Dolna Barycz R.	51°36'59.1"N	16°30'49.1"E	Holocene	4	27	2	4
total						5	36	3	8
**EN**	Bulgaria	Jantra R.	43°09'58.0"N	25°55'53.8"E	hybrid clade I	2	22	1	1
	Romania	Danube R.	44°04'47.9"N	26°43'51.2"E	hybrid clade I	3	15	2	9
total						5	37	3	10
**EET**	Germany	Neisse R.	51°51'28.8"N	14°36'34.9"E	Holocene	1	12	1	8
	Poland	Polska Woda R.	51°31'17.0"N	17°30'07.0"E	Holocene	3	16	-	-
	Poland	Dolna Barycz R.	51°36'59.1"N	16°30'49.1"E	Holocene	2	5	1	2
	Czech Rep.	Pšovka Cr.	50°22'11.8"N	14°33'06.8"E	Holocene	1	2	-	-
total						7	35	2	10
**ETT**	Germany	Issel R.	51°51'00.0"N	06°15'00.0"E	Holocene	2	13	2	3
	Germany	Ilmenau R.	53°22'34.0"N	10°14'36.5"E	Holocene	1	2	-	-
	Czech Rep.	Laboratory B1	50°24'37.6"N	14°27'16.9"E	B1 generation	3	17	2	10
total						6	32	4	13
**EEN**	Bulgaria	Vit R.	43°15'47.0"N	24°19'30.1"E	hybrid clade I	2	56	2	6
	Poland	Polska Woda R.	51°31'17.0"N	17°30'07.0"E	hybrid clade I	1	7	1	1
	Romania	Barlat R.	46°40'23.8"N	27°40'07.5"E	Holocene	3	6	-	-
	Romania	Comana R.	44°10'07.1"N	26°08'54.9"E	hybrid clade I	1	2	-	-
	Romania	Adjud R.	46°04'21.0"N	27°12'32.2"E	Holocene	1	2	-	-
	Slovakia	Cierna voda R.	48°36'27.0"N	21°59'34.1"E	hybrid clade I	8	51	5	17
total						16	124	8	24
**ENN**	Romania	Danube R.	44°04'47.9"N	26°43'51.2"E	Holocene	10	35	3	12
total						10	35	3	12

Abbreviations: Capital letters represent sets of haploid genomes: E, *Cobitis elongatoides*; T, *C*. *taenia*; N, *C*. *tanaitica*. Shortcuts in the column captions: AOC, age of clones; NOI, number of individuals; NOMKA, number of metaphases karyologically analysed; NOGE, number of GISH experiments; NOAGM, number of analysed GISH metaphases.

In order to prepare probes for GISH experiments only gDNA was used from individuals marked with *.

### Ethics statement

All specimens were collected in accordance with the national legislation of the countries concerned. The experimental procedures involving fish were approved by the Institutional Animal Care and Use Committee of the Institute of Animal Physiology and Genetics, CAS, v.v.i, according with directives from State Veterinary Administration of the Czech Republic, permit number 217/2010, and by the permit number CZ 00221 issued by Ministry of Agriculture of the Czech Republic. LC has Certificate of competency according to §17 of the Czech Republic Act No. 246/1992 coll. on the Protection of Animals against Cruelty (Registration number: CZU 955/06), provided by Central Commission for Animal Welfare, which authorizes animal experiments in the Czech Republic.

### Chromosome preparation

Metaphase chromosomes were prepared according to Ráb and Roth [39] with slight modifications. Briefly, fish were injected with 0.1% colchicine solution (1 ml/100 g of body weight) 45 min before being sacrificed using an overdose of 2-phenoxyethanol anaesthetics agent (Sigma). The kidneys were removed, dissected in 0.075 M KCl at room temperature and the cell suspension free of tissue fragments was hypotonized for 8 min in 0.075 M KCl, fixed in fresh prepared fixative (methanol: acetic acid 3:1, v/v), washed twice in fixative, and finally spread onto slides. We also used a preparation of chromosomes from regenerated caudal fins according to Völker and Ráb [40].

### Karyotype analysis

**Fig 1:**
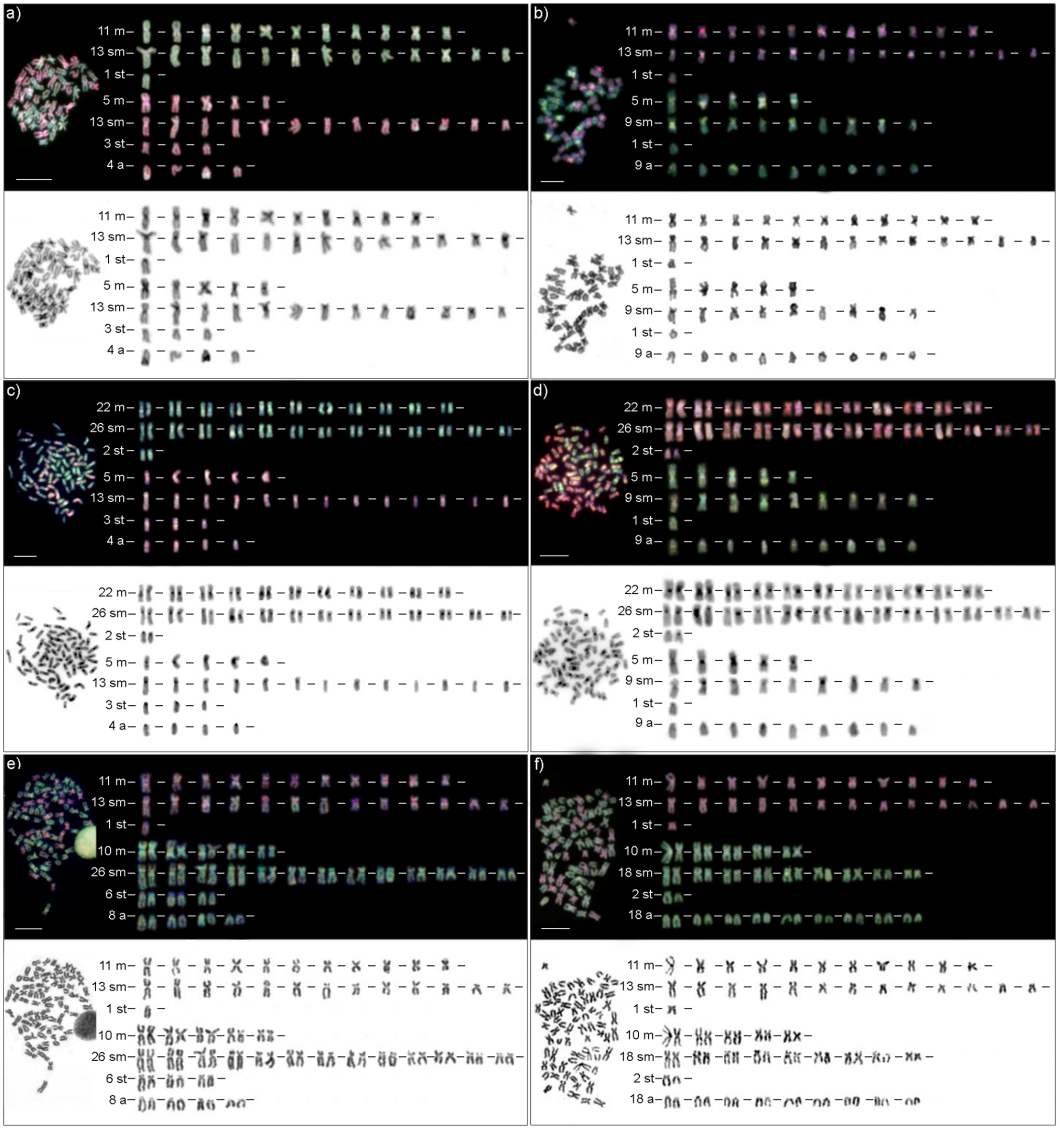
Representative karyotypes of hybrid biotypes after GISH and/or DAPI/Giemsa staining. (A) EN hybrid metaphase with hybridization pattern of *Cobitis elongatoides* gDNA in green, *C*. *tanaitica* gDNA in red. (B) ET hybrid with *C*. *elongatoides* in red, *C*. *taenia* in green. (C) EEN hybrid with *C*. *elongatoides* in green, *C*. *tanaitica* in red. (D) EET hybrid with *C*. *elongatoides* in red, *C*. *taenia* in green. (E) ENN hybrid with *C*. *elongatoides* in red, *C*. *tanaitica* in green. (F) ETT hybrid with *C*. *elongatoides* in red, *C*. *taenia* in green. Capital letters represent haploid genome sets: E, *C*. *elongatoides*; N, *C*. *tanaitica*; T, *C*. *taenia*. Chromosomes were arranged in a decreasing size order and classified in four morphological groups: metacentric (m), submetacentric (sm), subtelocentric (st) and acrocentric (a). Probes labelled with biotin-16-dUTP were detected with FITC-streptavidin (green signals on chromosomes); probes labelled with digoxigenin-11-dUTP were detected with anti-digoxigenin-rhodamin (red signals on chromosomes). To visualize the morphology of chromosomes DAPI (A, B, C, D) or Giemsa (E, F) stained karyotype was used. Captured DAPI stained karyotypes were inverted. Bars equal 5 μm. Detail information about individuals used is provided in S1 Table.

Metaphases of each individual were inspected by Giemsa or DAPI staining in order to confirm number and morphology of their chromosomes. In total 299 metaphases of 49 hybrids were inspected (Table 2). Metaphases were classified using the nomenclature proposed by Levan *et al*. [41] and classified into four categories: metacentrics, submetacentrics, subtelocentrics and acrocentrics. Subset of metaphases from 23 hybrid individuals was arranged in a decreasing size order to demonstrate consistency of karyotypes (Fig 1A–1F and S1A–S1R Fig, S1 and S2 Tables).

### Probe preparation, GISH

A subset of 23 hybrid individuals was used for further GISH analyses to determine whether the original parental chromosomal sets are distinguishable in hybrid karyotypes. At least two GISH experiments with different individuals were successfully performed *per* biotype (S1 and S3 Tables). Probes used in GISH experiments were prepared from whole genomic DNA (gDNA) of pure parental species *C*. *elongatoides*, *C*. *taenia* and *C*. *tanaitica*. gDNA was extracted from muscles or fins using the DNeasy Blood and Tissue Kit (Qiagen, Hilden, Germany) according to the manufacturer’s instructions. gDNA samples were labelled with biotin-16-dUTP (Roche, Mannheim, Germany) and digoxigenin-11-dUTP (Roche) using a Nick Translation Mix (Roche) following the protocol supplied by the manufacturer. The best results were obtained after 1.5–5 h (time strongly depends on the starting gDNA quality and fragment length) of nick translation until labelled DNA fragments were approximately 200–500 bp long. Species-specific hybridization probes combined gDNA of sexual species (*C*. *elongatoides* and *C*. *taenia*, or *C*. *elongatoides* and *C*. *tanaitica*) to perform GISH experiments on chromosomes of hybrids (Table 2). Salmon sperm was used as blocking reagent for repetitive DNA. The hybridization and detection procedure were carried out under conditions described by Symonová *et al*. [42]. The biotin-dUTP labelled probes were detected using streptavidin-FITC (Invitrogen, San Diego, Calif., USA). The digoxigenin-dUTP labelled probes were detected using anti-digoxigenin-rhodamin (Roche). The chromosomes were counterstained with Vectashield/DAPI (1.5 mg/ml) (Vector, Burlingame, Calif., USA).

### Image processing

Chromosomal preparations were examined by an Olympus Provis AX 70 epifluorescence microscope. Images of metaphase chromosomes were recorded with a cooled Olympus DP30BW CCD camera. The IKAROS and ISIS imaging programs (Metasystems, Altlussheim, Germany) were used to analyse grey-scale images. The captured digital images from GISH experiments were pseudocoloured (blue for DAPI, red for anti-digoxigenin-rhodamin, green for streptavidin-FITC) and superimposed using Microimage and Adobe Photoshop software, version CS5, respectively.

### Estimates of clonal ages and speciation times in mutational-time units using the coalescent methods

We have applied molecular clocks in order to evaluate the absolute time scale over which observed karyotype changes have accumulated among parental sexual species and their hybrids. For estimates of ages of clonal lineages we used cytoplasmic markers (mtDNA) because it is haploid and traces the clonal evolution to its original formation event. We note that estimation clonal ages from nuclear markers is much more complicated since nucleus of clones is of hybrid nature and subject of polyploidization through subsequent genome incorporations (‘leaky gynogenesis’ *sensu* Janko *et al*. [28]). On the other hand, for the estimation of parental species speciation time, nuclear markers (nDNA) had to be used since Choleva *et al*. [27] demonstrated massive mtDNA introgression from *C*. *elongatoides* to *C*. *tanaitica* suggesting its mtDNA diversity reflects secondary hybrid introgression rather than its speciation from sister species *C*. *taenia*.

Given the aforementioned necessity to use distinct sets of markers for divergence time estimates of clones and sexual species, respectively we performed two-step analysis to obtain, comparable time estimates from both types of markers. First (1), we applied coalescent method to independently estimate the clonal ages and speciation times on the relative time scale (expressed in mutational-time units that are scaled by locus-specific mutation rates). In a second step (2), we translated such estimates into absolute-time units by consistently calibrating both markers (mtDNA and nDNA) with the same outgroup species.

1) Coalescence analysis: The age of the oldest known *Cobitis* clonal lineage was estimated as the time to the most recent common ancestor (TMRCA) for all cyt *b* sequences of asexual individuals sampled by Janko *et al*. [26] that cluster in the so called ‘Hybrid clade I’. TMRCA was estimated by coalescent method of Griffiths and Tavare [43] and it was implemented in the Genetree software by R. C. Griffiths, which is particularly suitable because the observed pedigree of the Hybrid clade I conformed to the infinite-allele model (we observed no evidence for multiple mutational hits on sequence positions within this clade). We first determined the Maximum Likelihood (ML) value of *θ* (*θ =* 2*N*_*f*_μ for haploid maternally inherited mitochondrial locus; *N*_*f*_ represents the effective population size of females, and μ represents the mutation rate per locus per generation). Using the ML estimate of *θ* (1.156) we performed one million simulations of the coalescent process providing us with an estimate of TMRCA and 95% confidence intervals in coalescent units.

Speciation times in mutation units of *C*. *elongatoides*, *C*. *taenia* and *C*. *tanaitica* as well as their contemporary and ancestral population sizes, *θ*_TN_, *θ*_N_, *θ*_E_, *θ*_TNE_ and *θ*_TN_ (*θ =* 4*N*_*e*_μ) were estimated from sequences of nine nuclear loci from Choleva *et al*. [27]. The calculation was performed with the Bayesian Markov chain Monte Carlo (MCMC) algorithms implemented in BPP Version 2.0b [44], which allow for the incorporation of relative mutation rates among loci and multiple haplotypes per species. The analysis was performed under the assumption of (TT, NN) monophyly in the true species tree (see Choleva *et al*. [27]). As recommended in Yang [45], the mutation rate scalars (i.e. the information about the relative substitution rates of individual loci) were obtained by among-locus comparison of average distances between ingroup and outgroup species. As an outgroup we used the sexual species *C*. *paludica*, which is a phylogenetically distant taxon suggesting that the coalescent error (i.e. the deviations among loci caused by polymorphism within the common ancestor of in-group and outgroup taxa) would not be of a large magnitude [45]. With this outgroup the MCMC was run under conditions described in Choleva *et al*. [27].

2) In the second step, we converted abovementioned estimates into absolute-time units, which require a calibration point on *Cobitis* phylogeny that is suitable for employed nuclear markers. Since *Cobitis* phylogeny has been mostly addressed by mtDNA loci we first constructed an ultrametric tree of published cyt *b* sequences covering all major *Cobitis* lineages (dataset from Tang *et al*. [46]) using previously published calibration point, i.e. the opening of the Gibraltar strait (~5.33 MYA), which separated two sister species, i.e. *C*. *maroccana* and *C*. *paludica* [47]. Although the usage of this calibration point may have limitations due to its recency, obtained ultrametric tree provided two relevant facts: i) it allowed estimating the branch specific mutation rate of mitochondrial cyt *b*, which was used to convert relative estimates of clonal age into absolute time units, and ii) it provided an estimate of absolute divergence time between ingroup species and *C*. *paludica* outgroup. This time estimate was subsequently used to convert relative estimates of speciation times from nuclear markers into absolute time units and hence, both mtDNA and nDNA markers were calibrated consistently.

The data of Tang *et al*. [46] were used for ultrametric tree construction with two modifications. Firstly, we excluded sequences of taxonomically problematic species belonging to *Misgurnus* and *Paramisgurnus* genera (see Šlechtová *et al*. [48] discussion of this topic). Secondly, we replaced the *C*. *paludica* sequence from Tang *et al*. [46] with the *C*. *paludica* specimen from the Valle River, Spain (GenBank accession No: AY860180.1), as recommended by Doadrio and Perdices [47] because this population represents a phylogenetically closer lineage to the African *C*. *maroccana* and thus minimizes the coalescent error. Using such a modified dataset, we reconstructed the ML tree of cyt *b* sequences with RAxML [49] and ultrameterised it with the penalized likelihood method (PL) [50] implemented in R package Ape ([51]; S2 Fig). PL is a computationally inexpensive method that is nonetheless more robust against violations of model assumptions than strict-clock and relaxed-clock models [52]. ML estimates of branching times and branch-specific substitution rates depend on the lambda parameter which controls the variation of substitution rate change among branches (lambda may oscillate between zero -saturated model with one distinct rate for each branch—and infinity—model converging to a strict clock). Cross validation criterion removing one tip each time was used to select the optimal lambda value to construct the final ultrametric tree and obtain the ML estimates of branching times and substitution rates for each branch.

Having obtained the lineage-specific mutation rates, the TMRCA of the Hybrid clade I was translated into the time in years (*t*) as *t* = TMRCA*N_f_*g using the generation time (*g*) of two years (*N*_*f*_ was estimated from *θ* using the ML estimate of substitution rates provided by PL). In order to accommodate the uncertainty around mutation rate of clonal hybrids, which combine genetic material from two or more species, clonal age was estimated using an average rate over the branches leading to the species that participate in the origin of asexual *Cobitis* hybrids. Speciation times were translated into absolute time units by taking the ML estimate of the divergence time of *C*. *paludica* and ingroup species from the PL analysis.

## Results

### Karyotype analysis of hybrid metaphases

Giemsa stained metaphases of two diploid (EN, ET) and four triploid (EEN, ENN, EET and ETT) hybrid biotypes were analysed in order to evaluate the morphological stability of their karyotypes. Chromosomes were sorted out into one of four categories (meta-, submeta-, subtelo- and acrocentric chromosomes) based on their morphology. Without exception, all complete metaphases of all hybrid biotypes contained chromosomal numbers corresponding to appropriate combinations of haploid parental genomes involved, namely EN hybrid had 2n = 50, ET had 2n = 49, ENN and EEN had 3n = 75, ETT had 3n = 73, and EET had 3n = 74 (Fig 1, S1 Fig, Table 1). Also, numbers of chromosomes in particular morphological categories of all hybrids matched those expected by combining the karyotypes of parental species (e.g. in diploid hybrid of EN genomic constitution we observed 16 metacentrics– 11 metacentrics derived from E genome and 5 from the N genome; other categories could be derived similarly). These data are summarized in Table 1 and we further provide Giemsa or DAPI stained karyotypes of 23 individuals from all biotypes in Fig 1 and S1 Fig to document the consistency of such observations. Information about individuals used for karyotyping is given in S1 and S2 Tables.

We further focused on the differences among hybrids along the time gradient of their historical origin. We observed no detectable changes in karyotypes of hybrid clones irrespective of their age. In particular, Fig 1A and 1C, S1A–S1E Fig display representatives of ancient Hybrid clade I, Fig 1B and 1E, and S1F–S1O, S1Q and S1R Figs show representatives of lineage originated in the Holocene epoch; Fig 1D and 1F and S1P Fig shows the first generation hybrid obtained from recent laboratory crosses of parental species (S1 and S2 Tables).

### Identification of parental chromosomes in karyotypes of hybrid individuals based on GISH

Cytogenetic analyses were performed on metaphases of all 2n and 3n hybrid biotypes in order to identify parental chromosomal sets, and to detect potential large-scale recombinations and/or rearrangements. Reliability of conventional approach was validated by GISH analysis of first generation hybrids in which we clearly sorted karyotypes into haploid sets categorized after parental species (Fig 1D and 1F, S3H and S3L Fig). After having applied GISH to wild caught hybrids, we got the same result and clearly assigned chromosomes to haploid chromosomal sets of sexual species involved in the primary hybridizations (i.e., to *C*. *elongatoides*, *C*. *taenia*, or *C*. *tanaitica*; Fig 1 and S3 Fig). For example, in GISH experiment to EN hybrid metaphases, the *C*. *elongatoides* signals were green and *C*. *tanaitica* signals were red (Fig 1A). According to GISH signal ratio we observed 11 meta-, 13 submeta- and 1 subtelocentric chromosome in green colour. Number and morphology of these chromosomes correspond to haploid set of chromosomes of pure *C*. *elongatoides* (Table 1). The rest of 50 chromosomes from EN hybrid metaphase were coloured in red and contained 5 meta-, 13 submeta-, 3 subtelo- and 4 acrocentric chromosomes which exactly matched to the haploid chromosome number of pure *C*. *tanaitica* (Fig 1A, Table 1). Analogously, after GISH experiments with all other hybrid biotypes (Fig 1B–1F and S3 Fig), the chromosomes matched by numbers and by morphology to combination of haploid and diploid chromosome set of respective sexual species. The summary of these observations is provided in Table 1.

In concordance with observation from Giemsa stained metaphases no detectable differences in karyotypes were observed among hybrids of different age of origin (Fig 1, S3 Fig, S1 and S3 Tables). No visible evidence for large-scale intergenomic exchanges was observed in the diploid and triploid hybrid metaphases of hybrids under this study. In some metaphases we found parts of individual chromosomes with balanced signals from both parental species but such observation resulted from non-specific hybridization rather than from recombination events and was never confirmed in other metaphases from the same individual including the same slide. GISH hybridization pattern was not uniform over the whole chromosomal length. The hybridization signals were stronger in the pericentromeric regions, particularly in metacentric chromosomes and in the telomeric regions of many other chromosomes (Fig 1 and S3 Fig).

### Age estimates of studied clones and speciation times of parental species

**Fig 2:**
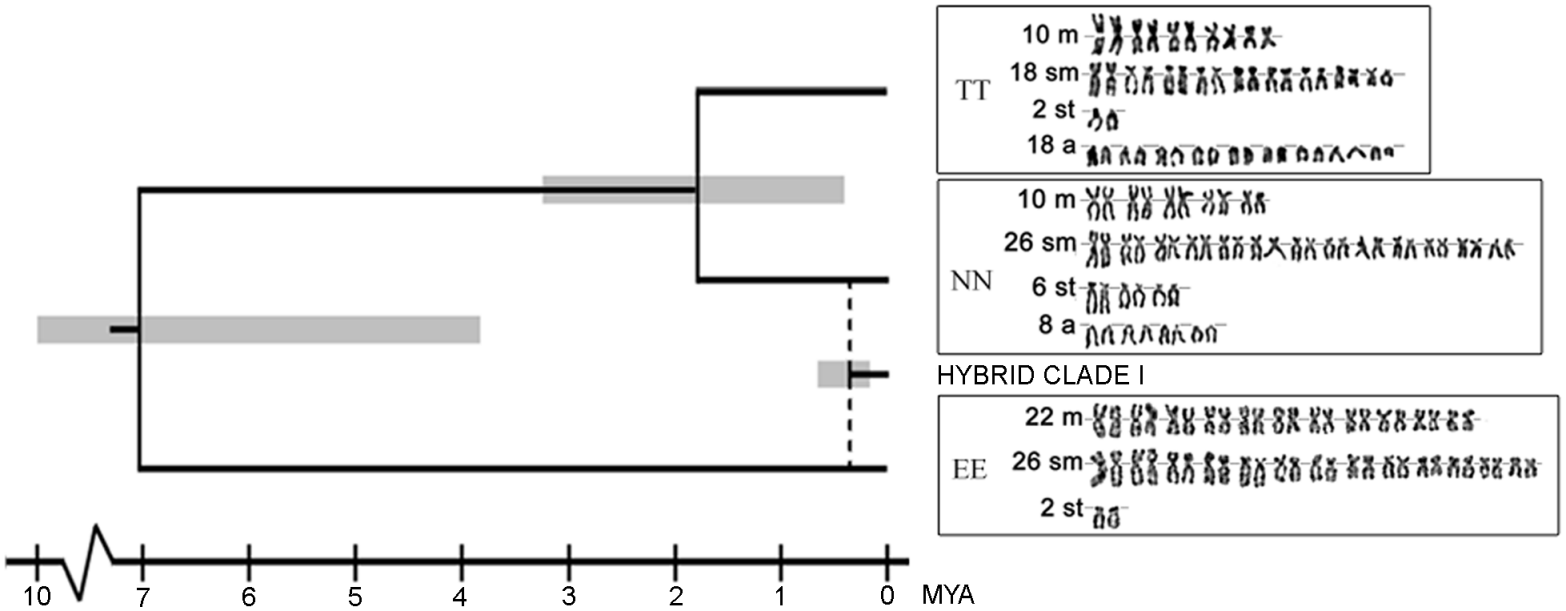
Ultrametric phylogenetic tree demonstrating estimated speciation times of parental species and the Hybrid clade I. Species-specific karyotypes arranged from Giemsa stained chromosomes are shown along the right side of cladogram. Confidence intervals of nodes of interest are in grey colour. TT, *C*. *taenia*; NN, *C*. *tanaitica*; EE, *Cobitis elongatoides*. Chromosomes were arranged in a decreasing size order and classified in four morphological groups: metacentric (m), submetacentric (sm), subtelocentric (st) and acrocentric (a).

The cyt *b* tree topology reconstructed with RAxML matched Tang *et al*. [46]. The cross-validation selected a relatively high lambda value (10^2^), which is consistent with Bohlen’s *et al*. [53] finding of no significant deviation from strict molecular clock in *Cobitis* cyt *b*. Using such a lambda value for PL method, we found relatively older cladogenetic events compared to Tang *et al*. [46] since these authors used a more distant *C*. *paludica* population as a putative sister lineage to *C*. *maroccana* for calibration, while we used a more closely related sequence from the Valle River, Spain (see [47]). The average cyt *b* substitution rate found by the PL method in the clade of hybridizing *Cobitis* species equalled 0.0065 per site per MYA. Having applied this rate to the ML estimate of the TMRCA of the Hybrid clade I provided a clonal age equal to 0.371 MYA (95% C I, 0.172–0.670 MYA) (Fig 2).

The ML estimate of a divergence time between *C*. *paludica* and the studied species within the *C*. *taenia* hybrid complex equalled 16.17 MYA. Using this divergence time as a calibration point for each nuclear locus from [27], the BPP software suggested that *C*. *elongatoides* diverged from the *C*. *taenia*–*C*. *tanaitica* ancestor during Miocene about 7 MYA (3.83–10.28 MYA), while both latter species diverged from each other for the period of Pleistocene-Pliocene about 1.8 MYA (0.42*–*3.25 MYA) (Fig 2).

Altogether, the results represent the hybrid biotypes under this study as representatives of a wide gradient of evolutionary ages (Table 2). The youngest hybrids are clearly represented by the first-generation individuals arisen by laboratory crosses. mtDNA analysis further revealed that natural hybrids formed two types with respect to their age. First type of hybrids shared haplotypes with the maternal species, demonstrating their recent origin during the Holocene epoch (see [26,30] for details). Second type of natural hybrids possessed haplotype E29 or E30 (*sensu* Janko *et al*. [26]) representing the oldest known *Cobitis* clonal lineage, the Hybrid clade I (Table 2).

## Discussion

### Stability of parental chromosomes in hybrid metaphases and their identification based on GISH

Analyses of Giemsa stained metaphases of hybrid clones enabled sorting of all chromosomes into morphological categories whose counts corresponded to expected numbers upon combining haploid complements of parental species. These observations were confirmed by GISH because chromosomal staining by species specific probes allowed unambiguous distinguishing of parental components in genomes of all studied hybrids. The observed pattern shows that chromosomes of hybrids did not undergo large-scale restructuring otherwise one would expect a failure to assign them to the morphological categories corresponding to karyotypes of respective parental species. Karyotypes of particular biotypes were uniform, regardless of whether these were of recent or ancient origin (including the oldest known *Cobitis* clonal lineage—the Hybrid clade I). This indicates the long-term stability of karyotypes of these asexuals. Our findings are not trivial from several reasons. Application of GISH to identify parental components in hybrids of cold-blooded vertebrates often faced technical hurdles stemmed from their small sized chromosomes. Hence, only a few previous studies reported successful results allowing further interpretations ([25] and subsequent references on *Ambystoma* salamanders, [54–62]). Moreover, our findings contrast other asexual organisms, e.g. *Sitobion miscanthi* where extra chromosomal element (originated by fission) was observed after seven years or less in laboratory culture [13].

Although various cytological mechanisms are supposed to maintain the long-term integrity of clonally inherited chromosomes, asexual organisms may have so called ‘minimal sex’ (i.e. occasional incorporation of low amount of alien genetic material into ova [63]) or sometimes switch between phases of sexual and asexual reproduction [64]. Such ‘sexual periods’ are expected to produce recombinant patterns of hybrid asexuals lineages, which were not observed in *Cobitis* hybrids studied here. Moreover, asexual transmission of genomes is seldom perfect, especially in automictic asexual lineages where limited recombinations between co-inherited genomes have been found (e.g. [25,63]). This may lead to a notable introgression of one parental genome onto the genomic background of the other one. Such intragenomic recombinations have the consequence in less heterozygous progeny. Arai and Mukaino [65] found that genetically variable progeny may also be formed by automictic organisms with premeiotic endoduplication and attributed such an observation to occasional formation of bivalents between the chromosomes from different parental taxa. Mitotic intraclonal gene conversion or recombination was also considered as one of possible explanations for exceptionally high rate of homozygosity in species-diagnostic SNP markers of hybrid clonal fish *Poecilia formosa* [66].

In contrast to aforementioned studies, this study showed that asexually transmitted *Cobitis* genomes maintain their integrity even in relatively old clonal lineages. We found no evidence of recombination between homeologous chromosomes from different parental species despite the fact that gametogenesis in hybrid *Cobitis* involves quasi normal meiosis [31,32]. Data from this and previous studies of *Cobitis* (see above) suggest that intraclonal recombinations among chromosomal complements originating from hybridizing parental species seem to be either absent or rare as they have not accumulated to a notable extent in hybrid clonal lineages whose ages span over hundreds of thousands of generations (Hybrid clade I). Although cytogenetic approach has a limitation in resolution power, there is possibility of detection of large-scale recombination as observed in unisexual salamanders [25]. On the other hand there is a case of *Pelophylax* hybrid complex where ongoing and site-specific interspecific genetic transfer mediated by hybridogenetic hybrids has been evidenced [63,67]. GISH experiments on metaphases of diploid hybrid *P*. *esculentus* which originate through hybridization between *P*. *ridibundus* and *P*. *lessonae* have detected two clear groups of chromosomal constituents, each originating from one of the sexual parental species but no intergenomic exchanges in metaphase chromosomes [54]. Similarly in *Cobitis* hybrid complex, if potential recombination occurred, these are small-scale changes and undetectable by conventional karyotype analyses or by GISH method. Our finding of integrity of karyotypes in *Cobitis* hybrids is well in line with previous cytological studies [31,32], experimental crossing experiments (e.g. [29]), and multilocus genotyping [28,30]. The result congruently indicates that *Cobitis* clones maintain a fixed heterozygosity in essayed species-specific loci. The only source of intra-lineage variation known so far stems from post formational mutations and incorporations of entire paternal genomes leading to increased ploidy of the clonally reproducing progeny. The integrity of clonal *Cobitis* genomes may theoretically be explained by high sequence divergence between parental species which prevents large-scale chromosomal recombinations. However, Bi and Bogart [25] described intergenomic recombination blocks on chromosomes of *Ambystoma* hybrids composed of *A*. *laterale* and *A*. *jeffersonianum* parental species. Despite authors did not provide divergence time of these species, Robertson *et al*. [68] showed that their divergence in cyt *b* (~ 10%) is more than twice as large than between *A*. *laterale* and *A*. *texanum* (~ 4%), which diversified about 10 MYA [69]. Recombination can therefore occur between genomes of species that are even more diverged than *Cobitis* under study (~ 7 MYA). Stability of Cobitid clonal genomes may, therefore, have another explanation.

### Is there an evidence for increased rates of chromosomal evolution?

The genetic variability of extant asexuals is derived from the variability possessed by their sexual progenitors at the time of primary hybridization. It means the variability of sexual genomes became frozen in forming clonal lineages. The clonal progeny observed nowadays thus bears the combination of genomes derived from the first generation of an interspecific hybrid. Therefore, our finding that parental chromosomal complements in clones exactly match morphological categories of karyotypes in contemporary sexual relatives suggests that neither the karyotypes of clones, nor the karyotypes of sexual species have accumulated morphological changes since the origin of hybrid lineages sampled in natural populations.

According to the molecular dating, parental species diverged from each other during Miocene and Pleistocene, respectively. Since then, their karyotypes differentiated in both number and categories of chromosomes [34,36] (Fig 2, Table 1) including the large changes in the positions of centromeres (particularly dynamic regions of chromosomal evolution in most eukaryotes [70]), as well as centric fusion. We cannot distinguish whether these changes have accumulated gradually or have been acquired during or right after speciation events. However, taking into account the level of karyotype differentiation among sexual species and the fast mutation rates documented in some clonal organisms [13,14,16–19], one would expect to observe at least some morphologically visible mutations accumulated in karyotypes of the much older *Cobitis* Hybrid clade I (ca 0.37 MYA which represents over one fifth of the time since the separation of *C*. *taenia* and *C*. *tanaitica*). Instead of increased rates of karyotype evolution, our current data suggest that asexual karyotypes lacked any notable reorganization despite they have been clonally transmitted more than several hundreds of thousands of generations since the initial clonal formation.

Abovementioned molecular dating should be considered with a large grain of salt due to large confidence intervals around the age estimates and other well-known problems inherent in molecular clock dating. However, another point that strongly supports the conclusion that clonally transmitted karyotypes evolve conservatively over long periods comes from biogeographic data. These imply that the clonal Hybrid clade I has survived at least one and probably more interglacials in multiple separated refugee [26]. We compared Hybrid clade I individuals that were sampled from areas that belong to different Danubian refugial areas as identified by Janko *et al*. [26]. Here, samples from Bulgaria and Romania represented the Lower Danubian refugium, samples from Eastern Slovakia represented the Pannonian refugium, and samples from Poland aimed to cover Central European/upper Danubian refugium (Table 2). Yet, we did not find karyotype differentiation among Hybrid clade I individuals originating from those distinct populations, which most likely evolved in isolation [26] suggesting that their identical karyotypes cannot be explained by homogenizing gene flow. Rather, we prefer the explanation that karyotypes of different refugial populations did not accumulate any morphological changes at least since the last interglacial.

We are aware that our conclusions are valid only for the resolution power of applied methods and sample number. Also, we may not exclude the accumulation of small-scale chromosomal mutational changes. For example, we observed indications of the accumulation of heterochromatin in the pericentromeric region in hybrid karyotypes. The phenomenon of the stronger signals in the centromeric regions is similar to the one described in *Ambystoma* salamanders [25], *Pelophylax* water frogs [54], and *Brassica* plants [71]. This may be explained by a higher degree of homeology of repetitive sequences on chromosome arms than at the centromeres with consequent weak blocking by the competitor DNA and a higher concentration of species specific repetitive sequences at centromeres [71]. Further investigation of the nature of karyotypic variation and genome evolution in *Cobitis* fishes will require the isolation and characterization of the major repetitive DNA sequences in the heterochromatin of each species to determine how homogeneous they are, and to map the sites of particular sequences on chromosomes by *in situ* hybridization. The same applies for mobile genetic elements such as retrotransposons, which are expected to degenerate in asexuals with the absence of meiosis [72,73], but there is nothing known about asexuals with modified meiosis, as for example in *Cobitis* hybrids.

## Conclusion

Conventional karyological studies of apomictic parthenogens show that asexuality increases the rate of chromosomal evolution [13,14,16,18] and could lead to accumulation of visible chromosome changes on short evolutionary time scale [13]. We applied conventional as well as molecular methods on karyotypes of automictic *Cobitis* hybrids and observed no morphological structural changes even in longer time scales. This indicates an interesting possibility that the effect of asexuality on the rate of chromosomal evolution is rather context-dependent than universal, i.e. it may depend e.g. on particular type of asexual reproduction. Future studies should take this possibility explicitly into account.

## Supporting Information

S1 Fig Representative karyotypes of hybrid biotypes after DAPI/Giemsa staining. (A-C) EN hybrids. (D-F) EEN hybrids. (G-I) ENN hybrids. (J-L) ET hybrids. (M-O) EET hybrids. (P-R) ETT hybrids. Chromosomes were arranged in a decreasing size order and classified in four morphological groups: metacentric (m), submetacentric (sm), subtelocentric (st) and acrocentric (a). To visualize the morphology of chromosomes DAPI (K and Q) or Giemsa (A-J, L-P and R) stained karyotype was used. Captured DAPI stained karyotypes were inverted. Bars equal 5 μm. Detail information about individuals used is provided in S2 Table.
(TIF)

S2 Fig Maximum likelihood phylogenetic tree of *Cobitis* and *Sabanejewia* species. ML phylogenetic tree constructed from cytochrome *b* gene sequences from Tang *et al*. [46] and from Doadrio and Perdices [47]. Calibration point follows Doadrio and Perdices [47]. Widths of branches are proportional to ML estimate of substitution rate. *Cobitis* species are highlighted (grey colour), species mentioned in this study are highlighted.
(TIF)

S3 Fig Representative metaphases of hybrid biotypes after GISH experiments. (A, B) EN hybrids. (C, D) EEN hybrids. (E, F) ENN hybrids. (G, H) ET hybrids. (I, J) EET hybrids. (K, L) ETT hybrids. Probes labelled with biotin-16-dUTP were detected with streptavidin-FITC (green signals on chromosomes); probes labelled with digoxigenin-11-dUTP were detected with anti-digoxigenin-rhodamin (red signals on chromosomes). Bars equal 5 μm. Detail information about individuals and hybridization patterns is provided in S3 Table.
(TIF)

S1 Table Hybrid individuals used for GISH experiments presented in Fig 1. (DOCX)

S2 Table Hybrid individuals used for karyotyping presented in S1 Fig. (DOCX)

S3 Table List of hybrid individuals used for GISH experiments presented in S3 Fig. (DOCX)
