# Supplementary figures and images for: Asexual Reproduction Does Not Apparently Increase the Rate of Chromosomal Evolution: Karyotype Stability in Diploid and Triploid Clonal Hybrid Fish (Cobitis, Cypriniformes, Teleostei)
Source: PLoS One. 2016 Jan 25;11(1):e0146872. doi: 10.1371/journal.pone.0146872 (PMC4726494; doi:10.1371/journal.pone.0146872)

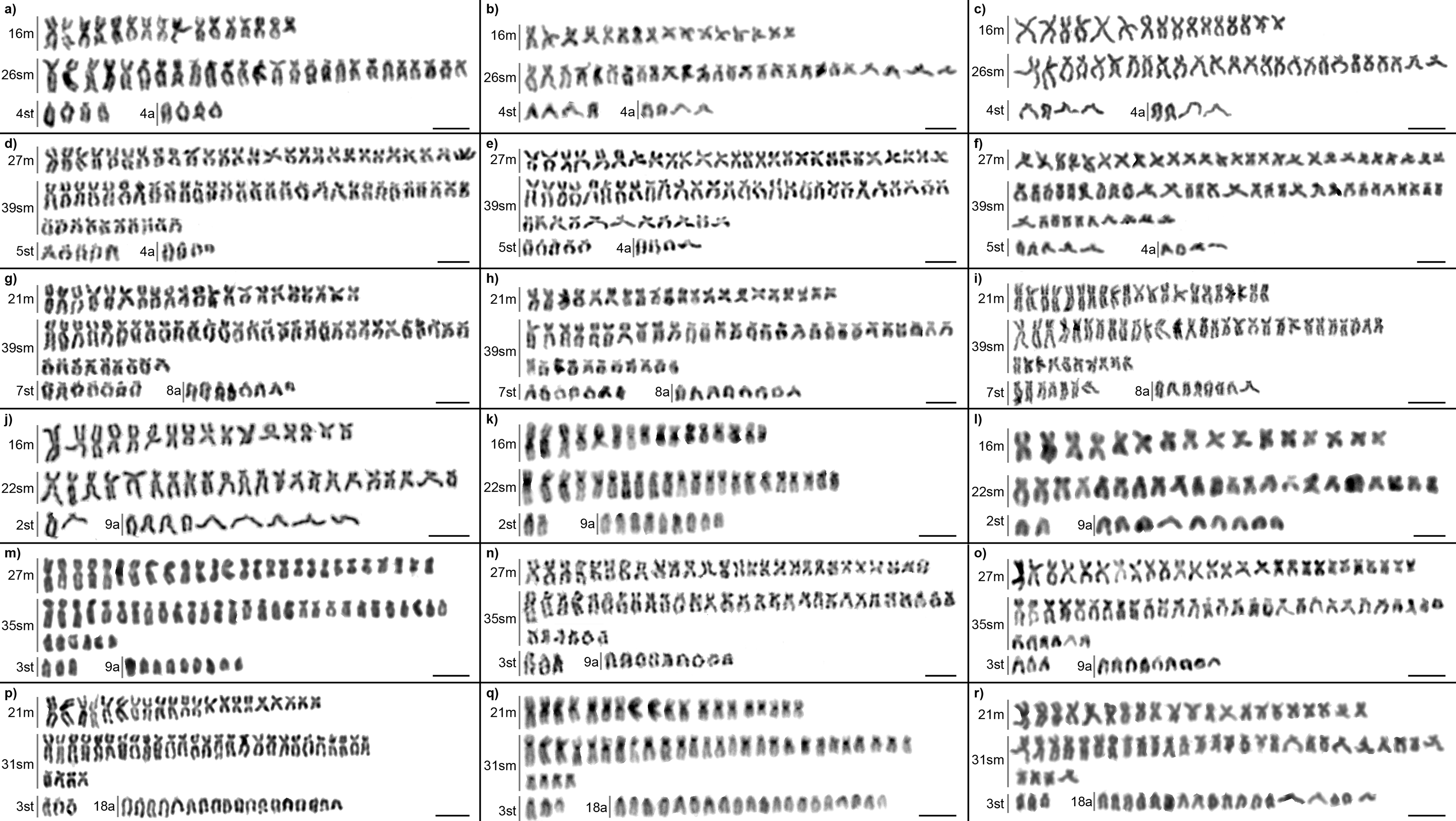

Supplement: S1 Fig — (A-C) EN hybrids. (D-F) EEN hybrids. (G-I) ENN hybrids. (J-L) ET hybrids. (M-O) EET hybrids. (P-R) ETT hybrids. Chromosomes were arranged in a decreasing size order and classified in four morphological groups: metacentric (m), submetacentric (sm), subtelocentric (st) and acrocentric (a). To visualize the morphology of chromosomes DAPI (K and Q) or Giemsa (A-J, L-P and R) stained karyotype was used. Captured DAPI stained karyotypes were inverted. Bars equal 5 μm. Detail information about individuals used is provided in S2 Table. (TIF) [file pone.0146872.s001.tif]

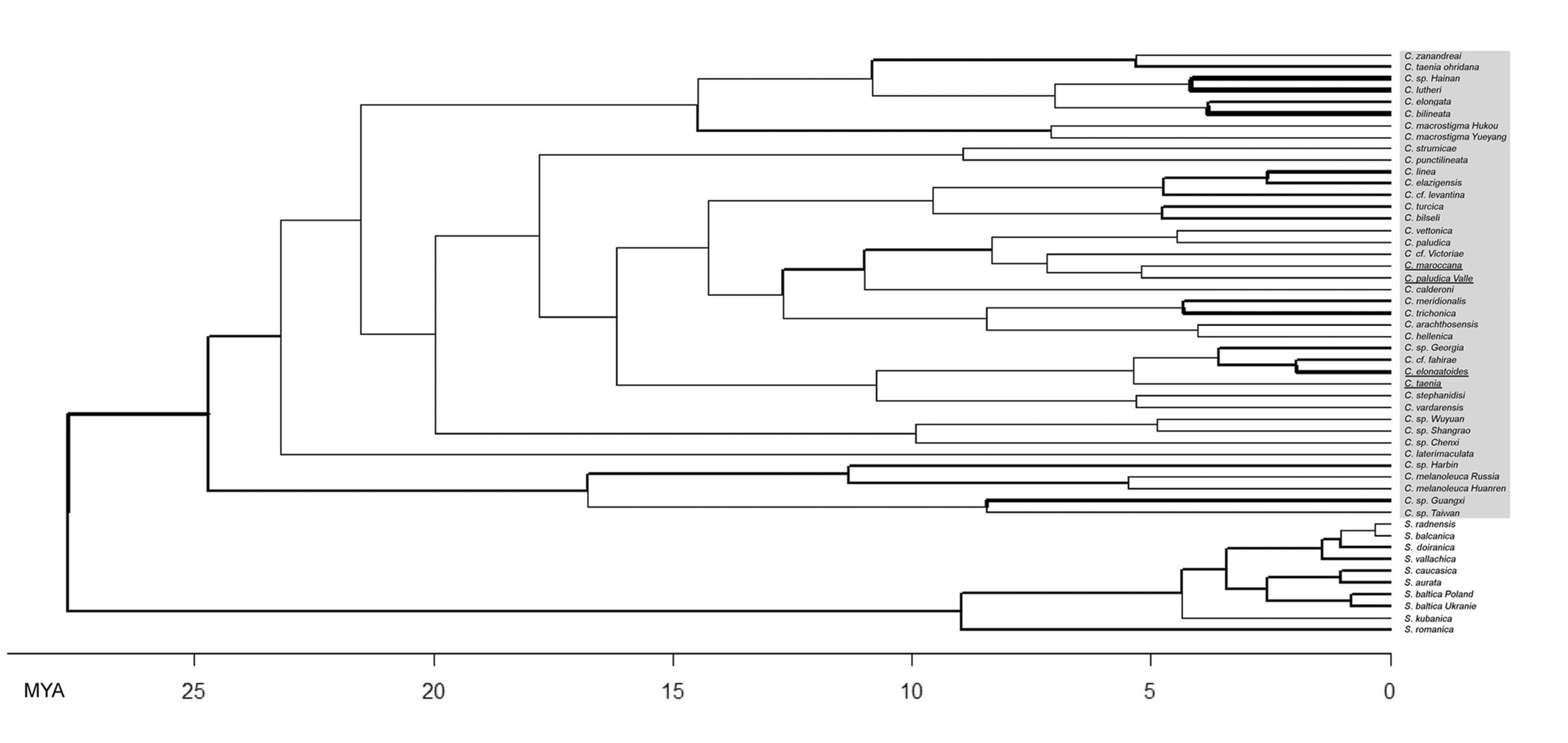

Supplement: S2 Fig — ML phylogenetic tree constructed from cytochrome b gene sequences from Tang et al. [46] and from Doadrio and Perdices [47]. Calibration point follows Doadrio and Perdices [47]. Widths of branches are proportional to ML estimate of substitution rate. Cobitis species are highlighted (grey colour), species mentioned in this study are highlighted. (TIF) [file pone.0146872.s002.tif]

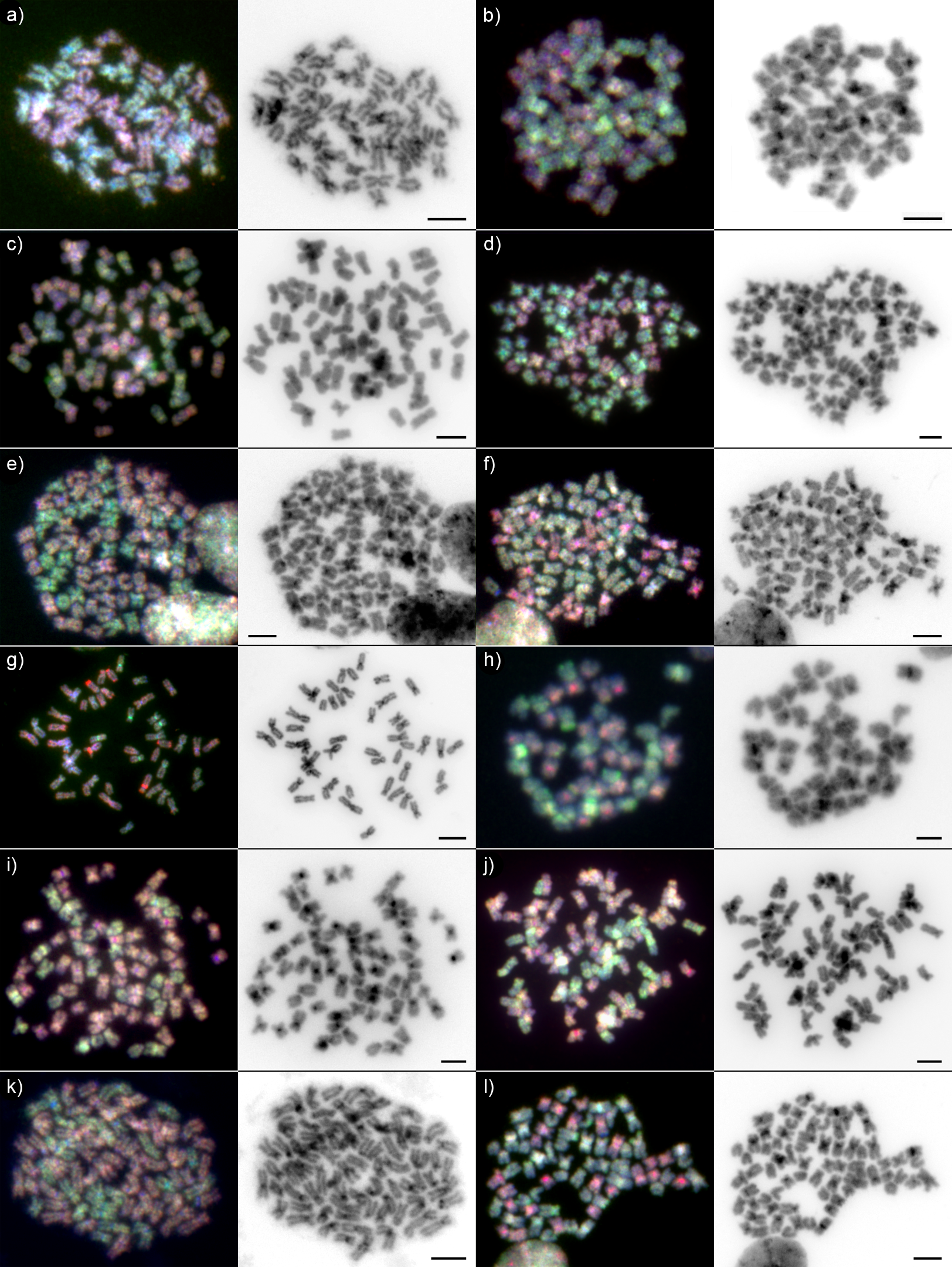

Supplement: S3 Fig — (A, B) EN hybrids. (C, D) EEN hybrids. (E, F) ENN hybrids. (G, H) ET hybrids. (I, J) EET hybrids. (K, L) ETT hybrids. Probes labelled with biotin-16-dUTP were detected with streptavidin-FITC (green signals on chromosomes); probes labelled with digoxigenin-11-dUTP were detected with anti-digoxigenin-rhodamin (red signals on chromosomes). Bars equal 5 μm. Detail information about individuals and hybridization patterns is provided in S3 Table. (TIF) [file pone.0146872.s003.tif]
