# Supplementary material for: Asexual Reproduction Does Not Apparently Increase the Rate of Chromosomal Evolution: Karyotype Stability in Diploid and Triploid Clonal Hybrid Fish (Cobitis, Cypriniformes, Teleostei)
Source: PLoS One. 2016 Jan 25;11(1):e0146872. doi: 10.1371/journal.pone.0146872 (PMC4726494; doi:10.1371/journal.pone.0146872)
Supplement: S1 Table — (DOCX) [file pone.0146872.s004.docx]

**S1 Table. Hybrid individuals used for GISH experiments presented in Fig 1**

|  | Biotype | Individual ID | Country | Locality | Lat | Long | Age of clone | Hybridization pattern |
| --- | --- | --- | --- | --- | --- | --- | --- | --- |
| a) | EN | EN1 | Romania | Danube R. | 44°04'47.9"N | 26°43'51.2"E | hybrid clade I | E - green, N - red |
| b) | EEN | EEN1 | Bulgaria | Vit R. | 43°15'47.0"N | 24°19'30.1"E | Holocene | EE - green, N - red |
| c) | ENN | ENN1 | Romania | Danube R. | 44°04'47.9"N | 26°43'51.2"E | hybrid clade I | E - red, NN - green |
| d) | ET | ET1 | Czech Rep. | Laboratory F1 | 50°24'37.6"N | 14°27'16.9"E | F1 generation | E - red T - green |
| e) | EET | EET1 | Germany | Niesse R. | 51°51'00.0"N | 6°15'00.0"E | Holocene | EE - red, T - green |
| f) | ETT | ETT1 | Czech Rep. | B1 generation | 50°24'37.6"N | 14°27'16.9"E | B1 generation | E - red, TT - green |

Abbreviations: Capital letters represent sets of haploid genomes: E, *Cobitis elongatoides*; T, *C. taenia*; N, *C. tanaitica*.
