# Supplementary material for: Asexual Reproduction Does Not Apparently Increase the Rate of Chromosomal Evolution: Karyotype Stability in Diploid and Triploid Clonal Hybrid Fish (Cobitis, Cypriniformes, Teleostei)
Source: PLoS One. 2016 Jan 25;11(1):e0146872. doi: 10.1371/journal.pone.0146872 (PMC4726494; doi:10.1371/journal.pone.0146872)
Supplement: S2 Table — (DOCX) [file pone.0146872.s005.docx]

**S2 Table. Hybrid individuals used for karyotyping presented in S1 Fig**

|  | Biotype | Individual ID | Country | Locality | Lat | Long | Age of clone |
| --- | --- | --- | --- | --- | --- | --- | --- |
| a) | EN | EN2 | Bulgaria | Jantra R. | 43°09'58.0"N | 25°55'53.8"E | hybrid clade I |
| b) | EN | EN3 | Bulgaria | Jantra R. | 43°09'58.0"N | 25°55'53.8"E | hybrid clade I |
| c) | EN | EN4 | Bulgaria | Jantra R. | 43°09'58.0"N | 25°55'53.8"E | hybrid clade I |
| d) | EEN | EEN2 | Slovakia | Cierna voda R. | 48°36'27.0"N | 21°59'34.1"E | hybrid clade I |
| e) | EEN | EEN3 | Slovakia | Cierna voda R. | 48°36'27.0"N | 21°59'34.1"E | hybrid clade I |
| f) | EEN | EEN4 | Bulgaria | Vit R. | 43°15'47.0"N | 24°19'30.1"E | Holocene |
| g) | ENN | ENN2 | Romania | Danube R. | 44°04'47.9"N | 26°43'51.2"E | Holocene |
| h) | ENN | ENN3 | Romania | Danube R. | 44°04'47.9"N | 26°43'51.2"E | Holocene |
| i) | ENN | ENN4 | Romania | Danube R. | 44°04'47.9"N | 26°43'51.2"E | Holocene |
| j) | ET | ET2 | Germany | Issel | 51°51'00.0"N | 6°15'00.0"E | Holocene |
| k) | ET | ET3 | Poland | Dolna Barycz R. | 51°36'59.1"N | 16°30'49.1"E | Holocene |
| l) | ET | ET4 | Poland | Dolna Barycz R. | 51°36'59.1"N | 16°30'49.1"E | Holocene |
| m) | EET | EET2 | Poland | Polska Woda R. | 51°31'17.0"N | 17°30'07.0"E | Holocene |
| n) | EET | EET3 | Czech Rep. | Pšovka Cr. | 50°22'11.8"N | 14°33'6.8"E | Holocene |
| o) | EET | EET4 | Poland | Polska Woda R. | 51°31'17.0"N | 17°30'07.0"E | Holocene |
| p) | ETT | ETT1 | Czech Rep. | Laboratory B1 | 50°24'37.6"N | 14°27'16.9"E | B1 generation |
| q) | ETT | ETT2 | Germany | Issel R. | 51°51'00.0"N | 6°15'00.0"E | Holocene |
| r) | ETT | ETT3 | Germany | Ilmenau R. | 53°22'34.0"N | 10°14'36.5"E | Holocene |

Abbreviations: Capital letters represent sets of haploid genomes: E, Cobitis elongatoides; T, C. taenia; N, C. tanaitica.
