# Supplementary material for: Asexual Reproduction Does Not Apparently Increase the Rate of Chromosomal Evolution: Karyotype Stability in Diploid and Triploid Clonal Hybrid Fish (Cobitis, Cypriniformes, Teleostei)
Source: PLoS One. 2016 Jan 25;11(1):e0146872. doi: 10.1371/journal.pone.0146872 (PMC4726494; doi:10.1371/journal.pone.0146872)
Supplement: S3 Table — (DOCX) [file pone.0146872.s006.docx]

**S3 Table. List of hybrid individuals used for GISH experiments presented in S3 Fig**

|  | Biotype | Individual ID | Country | Locality | Lat | Long | Age of clone | Hybridization pattern |
| --- | --- | --- | --- | --- | --- | --- | --- | --- |
| a) | EN | EN1 | Romania | Danube R. | 44°04'47.9"N | 26°43'51.2"E | Holocene | E - green, N - red |
| b) | EN | EN5 | Romania | Danube R. | 44°04'47.9"N | 26°43'51.2"E | Holocene | E - red, N - green |
| c) | EEN | EEN5 | Poland | Polska Woda R. | 51°31'17.0"N | 17°30'07.0"E | Holocene | EE - red, N - green |
| d) | EEN | EEN6 | Slovakia | Cierna voda R. | 48°36'27.0"N | 21°59'34.1"E | hybrid clade I | EE - green, N - red |
| e) | ENN | ENN5 | Romania | Danube R. | 44°04'47.9"N | 26°43'51.2"E | Holocene | E - green, NN - red |
| f) | ENN | ENN1 | Romania | Danube R. | 44°04'47.9"N | 26°43'51.2"E | Holocene | E - red, NN - green |
| g) | ET | ET5 | Poland | Dolna Barycz R. | 51°36'59.1"N | 16°30'49.1"E | Holocene | E - green, T - red |
| h) | ET | ET1 | Czech Rep. | Laboratory F1 | 50°24'37.6"N | 14°27'16.9"E | F1 generation | E - red T - green |
| i) | EET | EET1 | Germany | Niesse R. | 51°51'00.0"N | 6°15'00.0"E | Holocene | EE - red, T - green |
| j) | EET | EET5 | Slovakia | Cierna voda R. | 48°36'27.0"N | 21°59'34.1"E | hybrid clade I | EE - red, T - green |
| k) | ETT | ETT4 | Romania | Danube R. | 44°04'47.9"N | 26°43'51.2"E | Holocene | E - green, TT - red |
| l) | ETT | ETT1 | Czech Rep. | Laboratory B1 | 50°24'37.6"N | 14°27'16.9"E | B1 generation | E - red, TT - green |

Abbreviations: Capital letters represent sets of haploid genomes: E, *Cobitis elongatoides*; T, *C. taenia*; N, *C. tanaitica*.
